# Effects of Partial Freezing and Superchilling Storage on the Quality of Beef: A Kinetic Modelling Approach

**DOI:** 10.3390/foods14152687

**Published:** 2025-07-30

**Authors:** Anjelina William Mwakosya, Graciela Alvarez, Fatou Toutie Ndoye

**Affiliations:** INRAE, UR FRISE, Université Paris−Saclay, F-92761 Antony, France; anjelina-william.mwakosya@inrae.fr (A.W.M.); graciela.alvarez@inrae.fr (G.A.)

**Keywords:** beef, superchilling, superchilled storage, kinetic modelling, meat

## Abstract

The current study explores the changes in beef quality following partial freezing and during superchilled storage, alongside chilled storage comparisons. Kinetic models were developed to predict changes in colour difference (∆E), thiobarbituric acid−reactive substances (TBARS), total volatile basic nitrogen (TVB−N), drip loss and firmness. Beef samples were partially frozen in an air blast freezer at −30 °C for 9 min prior to storage at −5 °C, −4 °C, −2.8 °C, −1.8 °C. Chilled beef samples were directly stored at 2 °C and 6 °C without partial freezing. All samples were stored for 21 days. The lightness (L*), redness (a*), yellowness (b*) and colour difference (∆E) were significantly lower in superchilled storage samples compared to chilled storage samples. The pH of beef samples increased gradually over time (*p* < 0.05). TBARS, TVB−N and drip loss increased while firmness decreased with the increase in storage time in both storage conditions (*p* < 0.05). Overall, beef quality was affected by both storage duration and temperature. Firmness followed the first order kinetic model; drip loss, TVB−N, TBARS and colour difference (∆E) fitted the zero−order kinetic model. Temperature dependence was adequately modelled using Arrhenius−type equation with the activation energy values of 110.111, 52.870, 68.553, 119.480, 47.301 kJ/mol for drip loss, firmness, TBARS, TVB−N and colour difference (∆E), respectively. The models demonstrated strong predictive performance, with RMSE and MAPE values within ±10%. The developed kinetic models successfully predicted quality changes within the −5 °C to 6 °C temperature range.

## 1. Introduction

Meat is a highly perishable product due to its high−water content and nutrient dense matrix [1,2], which creates favourable conditions for various microbial and enzymatic activities. Thus, its processing, preservation and quality management remain critical areas of interest in the field of food science in general. With the expected global annual GDP increase in 3.5% annually and 34% increase in world population by 2030, the demand for meat in the diet is anticipated to rise significantly reaching 255,877 metric tons in 2027 [3,4]. In this sense, there is a need of supplying people with enough quantity of good quality, safe and healthy meat. These demands have driven significant advancements in preservation technologies, especially those involving low−temperature storage which are mostly refrigeration and freezing. However, these technologies often come with challenges. For instance, refrigeration storage (at 0–4 °C) offers limited shelf life of up to a week or less [5,6,7,8], while freezing storage (at −18 °C) extends shelf life up to 24 months [9]. However, freezing leads to undesirable deteriorative changes due to formation of ice crystals which in turn causes irreversible changes in the quality and microstructure of food [10,11]. Moreover, freezing often involves energy-intensive processes, high operational costs, and contributes to environmental issues such as carbon emissions, global warming, and ozone depletion [12].

To address these challenges, innovative preservation technologies have been explored with superchilling emerging as the best sustainable alternative process without compromising the safety and quality of food. Superchilling is simply performed by reducing the temperature of the food product to 1–2 °C below its initial freezing point. The whole process of superchilling mainly consists of two stages: (1) Partial freezing, which ensure about 30−35% of initial water is frozen depending on time−temperature conditions and freezing rate. It is in this stage where a thin ice crystal layer forms on the surface of the product. (2) Superchilled storage; this involves keeping the food product at a temperature of 1–2 °C below its initial freezing point. At this point, the ice layer formed from the first stage will absorb heat from the centre part of the product which is not frozen and eventually reach equilibrium in the whole volume in terms of temperature and ice crystal distribution [13]. Additionally, superchilling minimizes the need for freezing and frequently thawing, thereby lowering energy consumption and subsequent environmental impacts [14].

Several studies have explored the effect of superchilled storage temperature and time on different quality attributes such as pH, colour, drip loss, lipid oxidation, TVB−N, etc., on several food product types [15,16,17,18,19]. However, all the authors mentioned examined the effect of only a single superchilled storage temperature on the quality of food. This limited scope does not fully take into accounts practical challenges encountered in real−life cold chain logistics, where temperature fluctuations are common and can significantly affect product quality [20].To date, few studies in the literature have extensively explored the effect of temperature fluctuations during superchilled storage on meat quality. For instance, ref. [21] reported a significant increase in water loss, total volatile basic nitrogen (TVB−N), total viable count, and lipid oxidation of pork loins and salmon fillets stored under fluctuating temperature conditions of −3.5 ± 2.0 °C, compared to a narrow range of −3.5 ± 1.0 °C. In contrast, no significant changes were observed for samples stored at a constant temperature group of −3.5 °C for 30 days of storage. Similarly, ref. [22] investigated the effects of temperature fluctuations groups; −3 °C, −3 ± 3 °C and −3 ± 5 °C on the quality of raw pork. They found that temperature variation significantly decreased water holding capacity and accelerated lipid oxidation by measuring the thiobarbituric acid−reactive substances (TBARS) content after 20 days of storage with more serious drip loss. A recent study by [23] investigated the effects of temperature fluctuations during superchilled storage of chicken breast meat. Their study revealed that fluctuating condition of −1.3 ± 0.5 °C significantly elevated drip loss by 64% at day 21 of storage and noticeable changes in colour and pH. In contrast, samples stored under a constant storage condition of −1.6 ± 0.1 °C showed only 20% increase in drip loss over the same storage duration. Generally, all studies emphasized on the importance of precisely controlling temperature during superchilled for preserving meat quality. However, a gap still remains in the literature, as investigations into the effects of fluctuating temperature conditions during superchilled storage on beef meat are scarce.

While numerous studies have addressed the frozen beef storage [24,25,26], to the best of our knowledge, no existing study has systematically assessed the effect of a continuous broad range of storage temperatures of superchilled range (−1.8 °C and −2.8 °C), mild-freezing (−4 °C and −5 °C) and chilling temperatures (2 °C and 6 °C) at the same time particularly under conditions simulating cold chain fluctuations on the quality evolution of beef meat. Moreover, none of the studies have explored the use of kinetic models to predict the changes in the quality parameters of food products in the mentioned temperature ranges. According to [27], it is crucial to know about the quality kinetics of food in order to ensure safety of food during storage. In this sense, there are a number of studies that have developed kinetic models for predicting the quality of meat particularly in chilled and frozen storage conditions. For instance, ref. [28] developed oxidation kinetic models for predicting the total aerobic count (TAC), thiobarbituric acid−reactive substances (TBARS), and total volatile basic nitrogen (TVB−N) for rabbit meat stored under temperatures of −4 °C, −12 °C and −18 °C. Additionally, ref. [29] also developed models for predicting shelf life of chilled pork. Similarly, ref. [30] utilized kinetic models for predicting proteolysis, tenderness, and microbial growth for beef as accelerated by freezing process.

The primary objective of this study was to develop and validate kinetic models capable of predicting the evolution of quality parameters in partially frozen beef meat during post-processing storage under superchilling conditions. To this end, the temporal changes in key quality indicators, namely TBARS, TVB-N, drip loss, colour (ΔE, L*, a*, b*), firmness, and pH were monitored over a 21-day storage period. A broad range of storage temperatures was investigated, including chilling (2 °C and 6 °C), superchilling (−1.8 °C and −2.8 °C), and mild freezing (−4 °C and −5 °C), to both enable comparative analysis and reflect realistic temperature variations that can be encountered along the cold chain. Part of the experimental data were then used to calibrate kinetic models for each quality parameter, while model validation was performed using both the remaining experimental data and relevant literature sources.

## 2. Materials and Methods

### 2.1. Sample Preparation and Chemicals

Beef tenderloin samples (psoas major muscle) were purchased from a local butchery (Antony, France), approximately 24 h postmortem. The samples were sourced from a 24-month-old *Bos taurus* cattle. They were characterized with less visible fat and connective tissue. Approximately two kilograms of meat was acquired for each batch, all purchased at two different times, sourced from different animals but from the same anatomical region (tenderloin). The sample was placed in sterile bags and transported to the laboratory by car (approximately 5 min) under ambient conditions. After receiving, the beef meat was immediately placed in the refrigerator at 2 °C for 1 h for temperature equilibration before partial freezing. For quality measurements, the samples were cut in cylindrical shapes (3 cm in diameter × 1.5 cm in height) using a sharp stainless-steel corer. The cutting was performed perpendicular to the muscle fibres [31]. For each quality parameter, three set of replicates were performed at each storage temperature and measurement time point. The approximate average mass for each replicate was 10.0 ± 1.2 g. In general, all experiments for quality were carried out in two different batches to ensure reproducibility of results. All analytical-grade chemicals used in the experiment were purchased from Sigma Aldrich (Paris, France).

### 2.2. Superchilling Process–Experimental Design

The superchilling process was performed in two steps: partial freezing and subsequent superchilled storage. Initially the samples were subjected to partial freezing in an air blast freezer (Bonnet Biostore Turbo M, Bonnet Neve, EPTA, Le Perray-en-Yvelines, France). After cutting, the samples were immediately put on an open tray placed inside the freezing cabinet operating at an air temperature of −32 °C and a heat transfer coefficient of 58 W/m^2^K. The partial freezing time was calculated using Planck’s equation (Equation (1)) based on an assumed ice volume fraction of 30% yielding a calculated duration of 9 min. The parameters P and Q were assumed according to Planck model of infinite cylindrical shape as 16 and 4, respectively [32].

(1) $$t_{pf}=\frac{\rho L_{0}}{T_{f}-T_{a}}\left(\frac{d}{Qh_{a}}+\frac{d^{2}}{Pk}\right)$$

**Figure 1:**
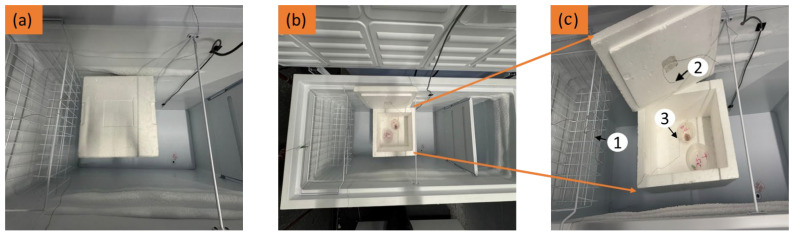
Storage and packaging setup for beef samples (**a**) Chest freezer interior with an expanded polystyrene (EPS) box (**b**) Overview of experimental storage layout (**c**) Close-up view of the expanded polystyrene (EPS) box containing closed plastic containers with beef samples inside, embedded with thermocouple for temperature monitoring. Temperature measuring points: (1) Air outside the EPS box (2) Air inside the EPS box (3) Internal product (beef) temperature.

Following partial freezing, the samples were immediately and equally put in six different polypropylene plastic containers (Amazon.fr, France) (each closed with snap-on plastic lids) and they were placed in a 5 cm thick expanded polystyrene box in order to achieve constant temperature. These boxes were then placed in four distinct chest freezers (Electrolux ECM30132W, Stockholm, Sweden) which were each set at a constant temperature: (i) −5 °C (ii) −4 °C (iii) −2.8 °C (iv) −1.8 °C (see Figure 1 for photographs of the packaging and storage setup). For the chilling condition, the cut samples were put directly in the polypropylene plastic containers (without partial freezing) and placed in a chiller set at 2 °C and 6 °C, respectively. The storage temperatures were potentially selected based on the measured initial freezing point of the beef (−0.89 °C), which was determined experimentally by extrapolating the plateau of the freezing curve as previously described by [33]. Therefore, the temperatures of −1.8 °C and −2.8 °C were specifically chosen for superchilled storage, as they fall within the superchilled range of approximately 1−2 °C points below the initial freezing point of beef. Consequently, other additional temperatures (−5 °C, −4 °C, 2 °C and 6 °C) were included to account for potential temperature fluctuations or cold chain break that can occur along the cold chain and subsequent effects on the quality of beef meat. For instance, higher temperatures (2 °C and 6 °C) represent chilling conditions that might possibly occur if storage systems fail to maintain superchilled condition and they were also used as control for comparison with superchilling conditions, while lower temperatures (−5 °C and −4 °C) simulate cases where the temperature might drop below the desired superchilled range. Chilling temperatures were studied to enable a comparison between superchilling and chilling conditions. The samples were analyzed for quality parameters before (fresh) and immediately after partial freezing and during storage where sampling was performed after every 7 days as t_1_ ‘day 1’, t_7_ ‘day 7’, t_14_ ‘day 14’, and t_21_ ‘day 21’.

### 2.3. Measuring of Temperature During Partial Freezing and Superchilling Storage

During partial freezing, both the external air temperature and sample temperature were recorded using calibrated thermocouples (T−type thermocouple of 0.2 mm diameter and 0.1 °C of precision) connected to a data logger system (TC−08 Data Logger, Pico Technology, Cambridgeshire, UK) which was connected to a computer for data acquisition. During storage, each of the chest freezers and chiller were equipped with electronic temperature controller (Eliwell WM961, Villeneuve La Garenne, France) for a desired temperature setting. Moreover, the temperature of the samples, temperature of air inside and outside the polystyrene box in the storage unit were recorded after every 5 s using T−type thermocouples connected to a data logger (34970A, Agilent HP, Santa Clara, CA, USA) which was linked to a computer for continuous data acquisition throughout the whole storage duration.

### 2.4. Characterization of the Quality Parameters

#### 2.4.1. Determination of Drip Loss

Drip loss was determined using a method previously reported by [34]. Following each 7 days storage interval, the samples were weighed using a weighing balance (Sartorius AG, Germany), then left for 12 h to thaw in a refrigerator set at 4 °C. Each sample was positioned on a grid within an individual plastic box measuring 10 cm × 10 cm × 7 cm that had contained a dry absorbent paper. Thawed samples were subsequently weighed alongside the wet of absorbent paper placed inside the container, allowing the measurement of drip loss. The containers were sealed to ensure no further moisture loss or gain during thawing. Measurements were carried out in triplicate. Drip loss was then calculated as the percentage of partially frozen weight as shown by Equation (2).

(2) $$Drip\;loss\;in\;(\%)=\frac{W_{w}-W_{d}}{W_{s}}\times 100$$

where

$W_{w}$ = Weight of wet absorbent paper

$W_{d}$ = Weight of dry absorbent paper

$W_{s}$ = Weight of partially frozen beef sample

#### 2.4.2. Texture Analysis

Texture measurement was performed instrumentally using a texture analyzer (Stable Micro Systems, TA. XT Plus, Godalming, UK) equipped with a load cell of 10 kg. A flat−ended cylindrical probe (type P100; 100 mm diameter) was used to evaluate the texture by measuring the peak force required to compress the sample. The thawed sample was carefully placed on the support base in such a way that the fibre orientation of the beef sample was perpendicular to the probe fitted, then the probe was pushed into the sample at a constant travelling speed of 2 mm/s compressing the sample to 30% of the sample height which is approximately 0.45 mm. At the end of each run test, the force−time graph was recorded and plotted by the Exponent software. The peak force of the thawed sample was expressed as N and it was defined as firmness [35]. The mean value was calculated as the average of three measurements.

#### 2.4.3. Measurement of pH

The pH was measured following the method described by [36] with slight modifications. Approximately 25 g of thawed beef samples, obtained by pooling material from three individual samples, were minced and mixed with 40 mL of distilled water. The mixture was homogenized (Ultra Turrax T25, IKA, Braun, Germany) at 6000 rpm for 60 s. The homogenates were used to measure pH using a handheld pH metre (model WTW 315i; WTW, Weilheim, Germany) with a wide range of 0.00–14.00 range and high resolution of 0.01 pH resolution. pH measurements were carried out at ambient temperature in three replicates.

#### 2.4.4. Total Volatile Basic Nitrogen (TVB−N) Compounds

TVB−N was measured using Conway microdiffusion method according to [32]. This method was originally reported by [37]. Initially, 25 g of minced beef sample was mixed with 75 mL of distilled water and homogenized at 3000 rpm for 5 min. Thereafter, 2 mL of HCl was added to the homogenate in order to bring down the pH to 5.2 followed by heating the mixture to 70 °C and slowly cooled at room temperature. The mixture was then filtered, and 2 mL of the filtrate was added to the outer compartment with 1 mL of saturated potassium carbonate. Subsequently, 2 mL of 0.01 M HCL was added into the centre compartment of the Conway dish. Thereafter, the Conway dish was covered with a glass plate and left at 37 °C for 2 h. Finally, HCl was then titrated with 0.01 N NaOH with 3 drops of methyl red indicator. The TVB−N values were expressed as mg/100 g sample. 

#### 2.4.5. Thiobarbituric Acid−Reactive Substances (TBARS) Content

TBARS were measured to evaluate the extent of lipid oxidation in beef samples using the method developed by [38] with slight modifications. Briefly, a sample of 10 g of minced beef was mixed with 20 mL of 20% trichloroacetic acid (TCA) (Sigma Aldrich) and homogenized at 3000 rpm for 2 min. The homogenate was then centrifuged at 6000 rpm for 15 min. The resulting supernatant was filtered to obtain 5 mL of filtrate, which was then combined with 5 mL of 0.02 mol/L of 2−thiobarbituric acid (TBA) solution. The mixture was heated in a boiling water bath for 20 min alongside a blank containing 5 mL of 20% TCA and 5 mL of TBA reagent. Finally, the samples were cooled, and the absorbance was measured at 532 nm using a spectrophotometer (Model 722, Jinghua Science & Technology Co., Ltd., Shanghai, China). TBARS value was calculated using the calibration equation given by [18].

#### 2.4.6. Colour Measurement

Colour measurements were performed on the surface of thawed beef samples using a handheld colorimeter (CM−700d Spectrophotometer; Konica Minolta., Osaka, Japan) with a D65 illuminator at a standard observer angle of d/8°and 11 mm port/viewing area. The instrument was calibrated using a white plate and a black plate provided with the instrument. The measurements were taken perpendicular to the muscle fibres and were expressed in terms of hunter CIELAB colour intensity parameters, L* (lightness, 0 = black, 100 = white), a* (redness and greenness) and b* (yellowness and blueness) [39]. To account for variability, colour measurements were performed at three different locations of each sample and at least three independent samples were measured in order to obtain the average. To assess the difference between fresh beef meat samples and those stored under superchilled and chilled conditions for 1, 7, 14 and 21 days, total colour differences (ΔE) were calculated using the Euclidian distance operator as shown by Equation (3).

(3) $$∆E=\sqrt{{\left(∆L\right)}^{2}+{\left(∆a\right)}^{2}+{\left(∆b\right)}^{2}}$$

### 2.5. Kinetic Analysis

#### 2.5.1. Kinetic Modelling for Quality Parameters

Quality changes in beef meat were modelled using a kinetic approach based on the assumption that changes depend solely on storage temperature and storage time.

Generally, the reaction rate for the kinetics of quality changes in food products is expressed as shown in Equation (4) [40]:

(4) $$\frac{dC}{dt}=kC^{n}$$

For zero order reaction (*n* = 0), Equation (4) is integrated to yield Equation (5)

(5) $$C-C_{o}=-kt$$

For first order reaction (*n* = 1), Equation (4) is integrated to yield Equation (6)

(6) $$\ln\;\frac{C}{C_{o}}=-kt$$

where *C* and $C_{o}$ are the concentration of a measured quality parameter at time t and at the initial time (*t* = 0), respectively, *t* is the storage time in days and *k* is the apparent reaction rate constant for quality changes under each storage condition. The value of *k* is estimated using the least square method based on the statistical fit of the slope from a linearized plot: of $C-C_{o}$ versus time for zero order kinetics and ln $\;\frac{C}{C_{o}}$ versus time for first order kinetics.

Drip loss, TVB−N, TBARS and colour difference (∆E) were described by the zero order equation (Equation (5)), while texture changes was modelled using the first order equation (Equation (6)).

#### 2.5.2. The Relationship Between Kinetic Rate and Storage Temperature

Arrhenius model Equation (7) [41] was typically used to describe the temperature dependence of degradation kinetic rate of selected beef quality parameters. Moreover, these quality parameters were calculated based on Arrhenius model and integrated as previously described by [42]. The storage temperature (T) in this equation is the temperature of the product recorded after every 5 s for each temperature condition over a period of 21 days of storage.

(7) $$\;k=k_{0}\;\exp\left[\frac{E_{a}}{R}\left(\frac{1}{T}\right)\right]$$

where $k_{0}$ is called pre−exponential constant factor, k is the kinetic rate constant, $E_{a}$ (J$\cdot {mol}^{-1}$) is the activation energy, *R* represents the universal gas constant (8.314 J${\cdot mol}^{-1}K^{-1})$ and T (K) is the storage temperature. The $E_{a}$ and $k_{0}$ values were obtained from a linear regression of natural logarithm of the rate constants *k* versus the inverse of the storage temperature. The quality changes in beef stored at 6 °C, 2 °C, −1.8 °C, −2.8 °C and −5 °C were used for model parameter (*E_a_* and *k_0_*) fitting. Experimental data obtained at −4 °C were set aside for model evaluation and validation.

#### 2.5.3. Validation of Kinetic Models

The temperatures used for model parameter fitting were selected to cover a broad range of storage conditions, including chilling (2 °C and 6 °C), superchilling (−1.8 °C and −2.8 °C), and mild freezing (−5 °C). This wide temperature range was chosen to ensure that the models could describe the kinetics of quality changes across diverse and realistic cold chain scenarios.

To validate the applicability of the kinetic models, the samples stored at an intermediate temperature (−4 °C) within the studied range was selected in order to avoid using extreme values. This approach for model validation is consistent with previous studies [43,44]. Therefore, the kinetic parameters ($E_{a}$ and *k_0_*) identified from experimental data obtained at the five other storage temperatures (6 °C, 2 °C, −1.8 °C, −2.8 °C, and −5 °C) were applied to predict the changes in quality parameter at −4 °C. The predicted results from the kinetic models were then compared with experimentally measured data at −4 °C for day 1, day 7, day 14 and day 21 of storage. Finally, the root mean square error (RMSE), as shown in Equation (8) and the mean absolute percentage error (MAPE) represented by Equation (9) were calculated for each quality parameter in order to evaluate the reliability, effectiveness and accuracy of the model predictions.

(8) $$RMSE=\sqrt{\frac{\sum_{i=1}^{N}\left({C_{obs}-C_{pre})}^{2}\right)}{N}}$$

(9) $$MAPE=\frac{100}{n}\sum \left|\frac{C_{obs}-C_{pre}}{C_{obs}}\right|$$

where $C_{obs}$ and $C_{pre}$ are the observed and predicted concentrations of quality parameter measured for each time point, respectively, and *n* is the number of observations.

### 2.6. Statistical Analysis

All measurements were run in triplicate and results are presented as mean ± standard error of means (SEM). The data were tested for normality using the Shapiro–Wilk test and homogeneity of variance via Levene’s test. Two−way analysis of variance (ANOVA) was conducted to analyze the effect of superchilled storage temperature and storage time and their interaction on the measured parameters. One−way ANOVA was conducted to analyze the variation among all measured parameters and Turkey’s post hoc test was applied to verify if there is a significant difference (*p* < 0.05) in samples. Additionally, an independent sample T−test was used to compare differences between fresh beef sample and sample taken immediately after partial freezing (partially frozen sample). All statistical analyzes were conducted using SPSS software package at a significance level of 95% (Statistical Package for the Social Science, version 19, SPSS Inc. Chicago, IL, USA). All graphs were obtained using GraphPad Prism 8 (Graph Pad Software, Inc. La Jolla, CA, USA).

## 3. Results and Discussion

### 3.1. Temperature Fluctuations During Superchilled Storage

**Figure 2:**
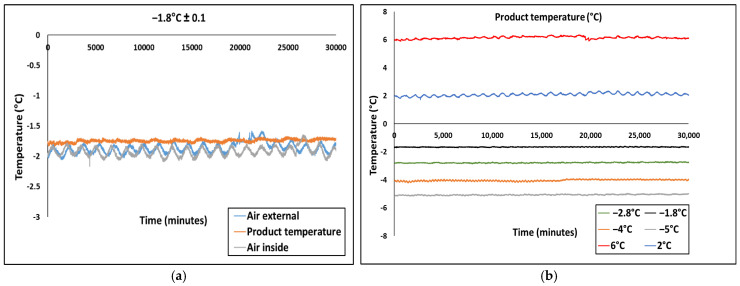
Time−temperature profile during storage. (**a**) Air and product temperatures at −1.8 °C superchilled storage. (**b**) Product temperature for both chilled and superchilled samples at six different storage conditions set, as shown in Table 1.

Figure 2a shows the temperature recordings of the beef meat sample stored at −1.8 °C as well as the air temperature inside and outside the polystyrene box throughout the storage period. Figure 2b presents the evolution of beef sample temperature for six distinct storage conditions investigated. It can be seen in both Figure 2a,b that the product temperature remained relatively constant at the set values with minor fluctuations. However, in Figure 2b, more fluctuations were noticeable for samples stored at 2 °C and 6 °C, respectively. This is because, the samples were placed directly at the top and bottom compartment without the EPS box, due to the limited size of the chiller. Similarly, in Figure 2a, slight and regular deviations were observed in the air temperature inside and outside the polystyrene box, which can be attributed to the on/off operation of the refrigeration system. A recent study by [22] reported a similar trend of fluctuations during superchilled storage of raw pork.

Table 1 provides the average product temperatures recorded for each condition throughout the entire superchilled storage duration. The data depicts that the insulation effect of both polystyrene and plastic boxes was sufficient to attenuate the temperature fluctuations more on samples stored at −1.8 °C, −2.8 °C, −4 °C and −5 °C than those stored at 2 °C and 6 °C with only plastic containers. Moreover, it is important to monitor product temperature, since it directly impacts the microstructure and consequently the overall quality of the beef meat.

### 3.2. Characterization of Quality Parameters of Beef

#### 3.2.1. Effect of Partial Freezing

**Figure 3:**
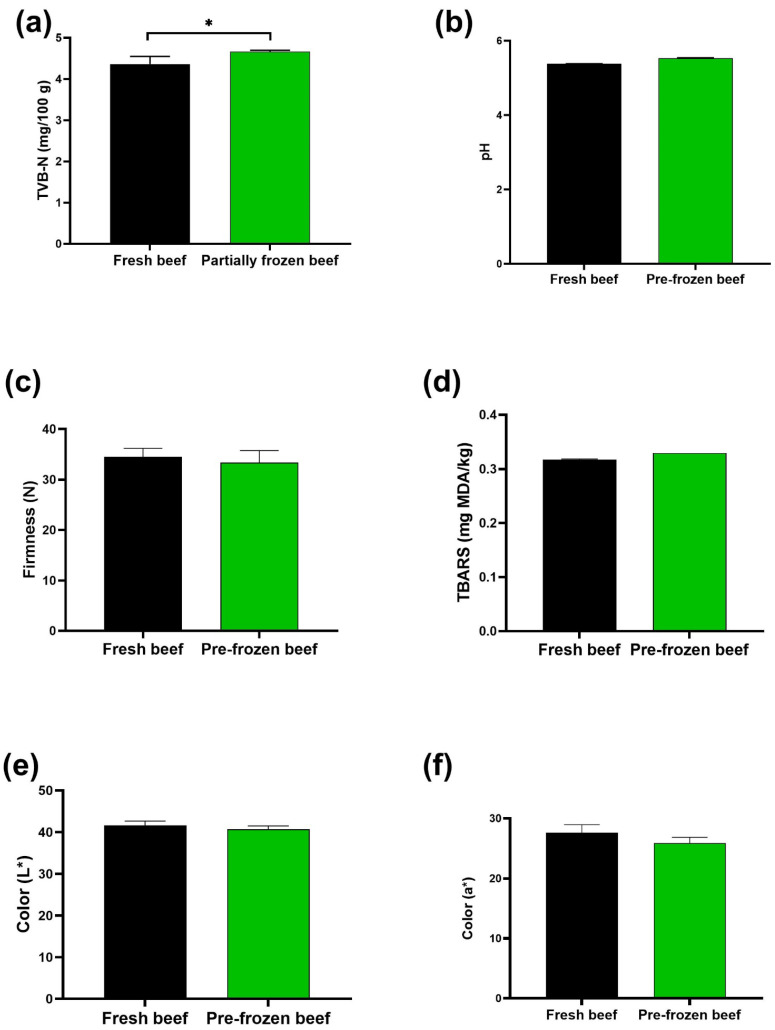
Effect of partial freezing on all measured quality parameters in fresh and pre-frozen (partially frozen) beef samples. (**a**) Total volatile basic nitrogen (TVB-N); (**b**) pH; (**c**) Firmness; (**d**) Lipid oxidation (TBARS); (**e**) Lightness (L*); (**f**) Redness (a*) values. The asterisk (*) above a line indicates significant differences between the groups. Values are means ± SEM (*n* = 3).

The effect of partial freezing on beef quality was evaluated by comparing various quality parameters measured in fresh and partially frozen beef. According to the results presented in Figure 3, no significant difference was observed in quality parameters including pH, TBARS, texture and colour (*p* > 0.05) suggesting that partial freezing did not have a substantial impact on these beef quality parameters. However, a significant increase in TVB−N (*p* < 0.05) was detected, indicating potential protein degradation at an early stage. This could be attributed by partial freezing or alternatively, the pre−existing contamination before partial freezing process. Previous studies have demonstrated that freezing can negatively affect the structural integrity and biochemical stability of proteins in beef meat [45,46]. As ice forms, the remaining unfrozen water becomes more concentrated with solutes such as salts and metabolites [47]. This increase in solute concentration enhances the ionic strength and lowers the pH of meat, thus destabilizing the initial structure of muscle proteins [48]. As a result, the structural proteins begin to partially unfold or denature even before complete freezing occurs.

#### 3.2.2. Drip Loss

**Figure 4:**
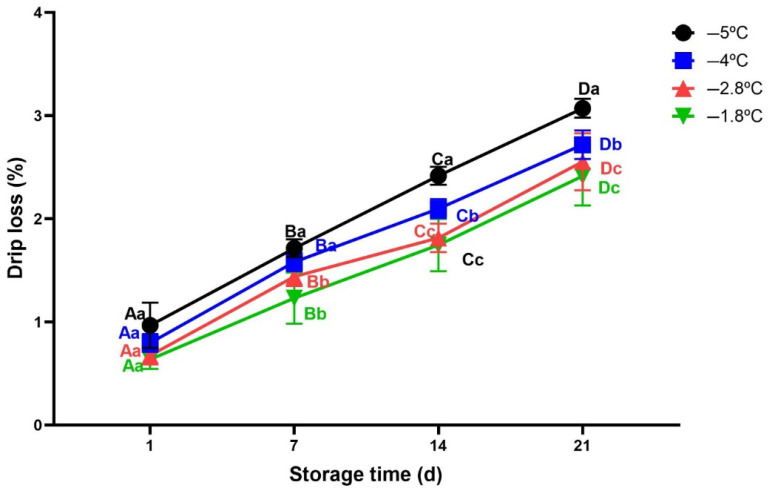
Variation in drip loss of beef meat during superchilled storage as a function of storage temperature. Error bars represent the standard error of the mean (SEM) calculated from three replicates (*n* = 3). Note: The data were expressed by the mean ± standard error of the mean. Different superscript lowercase letters in the same storage time indicated the significant difference (*p* < 0.05). Different superscript uppercase letters (A−D) in the same storage temperature indicated the significant difference (*p* < 0.05).

Drip loss of meat is undesirable because it leads to economic loss due to reduced product weight and negatively affects meat quality by making it drier and less tender [49]. In addition, drip loss causes loss of essential nutrients such as water soluble protein, vitamins and minerals [50,51]. As shown in Figure 4, drip loss increased significantly with the increase in storage time in all samples (*p* < 0.05). However, at day 1 of storage, there was no significant difference for all samples stored under different storage temperature conditions (*p* > 0.05). This is because at the beginning, the samples did not have enough time for storage temperature to affect water migration and crystal growth significantly. Over time, the beef samples stored at −1.8 °C and −2.8 °C, which had lower ice volume fractions (36% and 41%, respectively) exhibited significantly lower drip loss during storage compared to those stored at −4 °C and −5 °C (48% and 54%, respectively). The higher ice volume fractions at these lower temperatures typically resulted in larger ice crystal formation, causing extensive disruption of cellular integrity and increased water loss during thawing. The ice volume fractions was estimated based on the methodology described by [23]. In addition, [51] reported that a drip loss of 2% cannot be considered high for salmon and it should not be regarded as a major problem. However, in this study, drip losses higher than 2% were observed from day 14 of storage to day 21 of storage for beef samples stored at −4 °C and −5 °C. This phenomenon can also be explained due to ice recrystallization happening during extended storage. Over time, due to temperature fluctuations or storage duration, small ice crystals tend to melt and diffuse on the surface of larger ice crystals [23]. This process leads to increase ice crystals size thus causing permanent disruption of muscle cell membranes, leading to greater release of water duringthawing [52].

These findings prove that superchilling temperatures are effective for preserving the quality of beef meat. They also pose the importance of optimizing superchilled conditions in order to minimize drip loss and maintain the quality of beef along the cold chain.

#### 3.2.3. Texture Change

**Figure 5:**
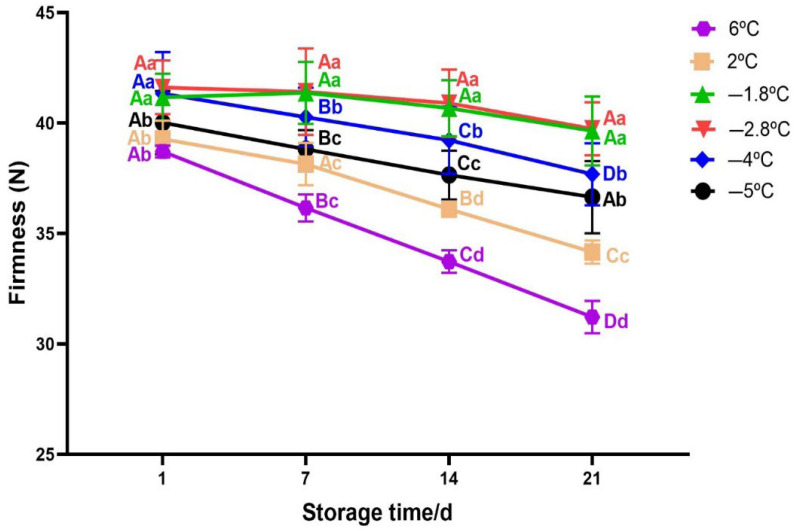
Variation in texture of beef meat during superchilled storage as a function of storage temperature. Error bars represent the standard error of the mean (SEM) calculated from three replicates *(n* = 3). Note: The data were expressed by the mean ± standard error of the mean. Different superscript lowercase letters in the same storage time indicated the significant difference (*p* < 0.05). Different superscript uppercase letters (A−D) in the same storage temperature indicated the significant difference (*p* < 0.05).

Beef texture is a paramount quality attribute since it directly influences consumer satisfaction [53]. In this study, texture was evaluated in terms of firmness which plays a significant role in determining the tenderness and overall palatability of beef [54]. In general, the firmness decreased with the increase in storage time (*p* < 0.05, Figure 5). Firmness of the sample depends on storage temperature and storage time as their interaction was significant (Two−way Anova; *p* < 0.05). On day 1 of storage, no significant differences in firmness were observed among samples stored at −5 °C, 2 °C, and 6 °C (*p* > 0.05). However, firmness values for these temperatures were significantly different from those of samples stored at −1.8 °C, −2.8 °C, and −4 °C (*p* < 0.05). Notably, no significant differences were found among the samples stored at −1.8 °C, −2.8 °C, and −4 °C (*p* > 0.05). Firmness decreased progressively with increasing storage duration; however, this reduction was not statistically significant at −1.8 °C and −2.8 °C (*p* > 0.05), suggesting enhanced textural stability at these temperatures. In contrast, firmness degradation was more pronounced at −4 °C and −5 °C than at −1.8 °C and −2.8 °C. The better preservation of textural integrity at −1.8 °C and −2.8 °C may be attributed to the reduced formation of large ice crystals, which helps maintain the microstructure and minimize damage to muscle fibres. On the other hand, lowest firmness values were observed in beef samples stored at 6 °C and 2 °C, with more decline at 6 °C. The trend observed in this study aligns with previous research [16,55]; they both explained that the reason for firmness decrease during storage is due to proteolytic activity by endogenous enzymes mostly in chilled samples. In addition, [56] correlated firmness with myofiber–myofiber attachment and they argued that weakening of these attachments resulted in a softer texture for superchilled samples.

#### 3.2.4. pH Measurement

**Figure 6:**
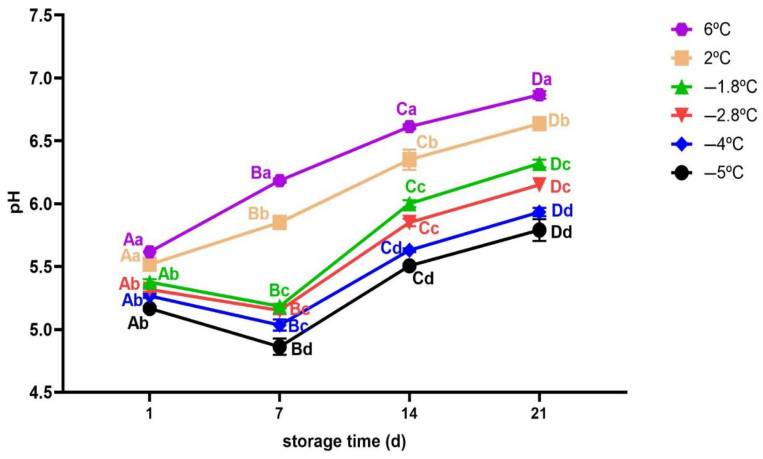
Variation in pH of beef meat during superchilled storage as a function of storage temperature. Error bars represent the standard error of the mean (SEM) calculated from three replicates (*n* = 3). Note: The data were expressed by the mean ± standard error of the mean. Different superscript lowercase letters in the same storage time indicated the significant difference (*p* < 0.05). Different superscript uppercase letters (A−D) in the same storage temperature indicated the significant difference (*p* < 0.05).

The changes in the pH values of beef meat during storage are shown in Figure 6. The initial value of pH was 5.38 ± 0.33. A similar result was also reported by [57] for a fresh beef. For beef samples stored at −1.8 °C and −2.8 °C, the pH decreased slightly to 5.3 and 5.2, respectively (*p* < 0.05). In contrast, samples stored at −4 °C and −5 °C showed significant decrease to 5.0 and 4.7, respectively (*p* < 0.05). A similar trend was reported for rabbit meat [18], pacific oyster [58], pork meat [59] and chicken breast meat [23]. The initial decrease in pH may be caused by accumulation of lactic acid and other solutes in the unfrozen portion of beef meat; since its only pure water that freezes [60]. This effect is more pronounced at lower temperatures such as −4 °C and −5 °C which correspond to higher ice volume fractions. The formation of more ice concentrates solutes in the unfrozen phase, consequently accelerating the acidification of meat during early storage.

However, the pH trend of samples stored at chilling temperatures (6 °C and 2 °C) exhibited a rapid and significant rise (*p* < 0.05) from day 7 onward, reaching 6.9 and 6.6, respectively, by day 21 of storage, as similarly reported by [15]. This may be mainly due to protein degradation caused by spoilage microorganism and endogenous enzymes which in turn leads to production of basic compounds such as free amino acids, ammonia, amines and other alkaline substances [18,61]. At the end of storage, the pH of superchilled samples at −5 °C and −4 °C was lower (*p* < 0.05) compared to the samples stored at −1.8 °C and −2.8 °C. However, the pH at −1.8 °C and −2.8 °C remained lower than the values observed for samples under chilling temperatures. This indicates that lower temperatures (−5 °C and −4 °C) are more effective in inhibiting microbial activity and enzymatic reactions, thereby reducing the rate of protein decomposition and slowing the rise in pH, whereas higher storage temperatures allow for increased activity leading to a more pronounced pH increase (*p* < 0.05). The present study found a significant interaction between storage temperature and storage time on pH of beef samples (Two-way Anova; *p* < 0.05).

#### 3.2.5. Changes in TVB−N of Beef During Storage

**Figure 7:**
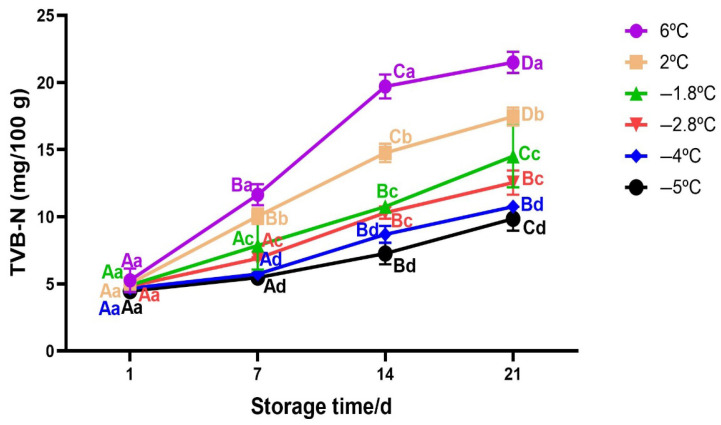
Variation in TVB−N of beef meat during superchilled storage as a function of storage temperature. Error bars represent the standard error of the mean (SEM) calculated from three replicates (*n* = 3). Note: The data were expressed by the mean ± standard error of the mean. Different superscript lowercase letters in the same storage time indicated the significant difference (*p* < 0.05). Different superscript uppercase letters (A−D) in the same storage temperature indicated the significant difference (*p* < 0.05).

TVB−N is used as an indicator for meat deterioration and it is composed of mainly basic compounds including ammonia and amines [62]. The accumulation of these alkaline compounds can affect beef odour and the shelf life of beef [59]. Changes in TVB−N during storage at different temperatures are shown in Figure 7. It can be seen that TVB−N values for all samples increased significantly with the increase in storage time (*p* < 0.05), consistent with previous studies on meat quality [55,63,64]. TVB−N of chilled samples stored at 2 °C and 6 °C exhibited higher rate of increase from 4.36 mg/100 g (initial value) to 16.52 mg/100 g and to 20.26 mg/100 g, respectively, over 21 days of storage. Contrary, for the same storage duration; superchilled samples exhibited a steady increase in up to 14.51 mg/100 g and 12.54 mg/100 g at −1.8 °C and −2.8 °C, respectively. These results align with previous studies by [55] on beef meat, where they obtained TVB-N values of 15.8 mg/100 g at day 21 for a superchilling storage of -1.5 °C. However, the increase in TVB-N was less pronounced at mild-freezing temperatures (−4 °C and −5 °C) reaching 10.75 mg/100 g and 9.86 mg/100 g, respectively, at day 21. A similar trend was reported by [59] on pork meat where at day 21 of storage, the TVB-N values of samples stored at −3 °C were lower compared to those stored at −1 °C and −2 °C, respectively. These results suggest that storage temperature and time are the most impacting factor of TVB−N values since their interaction was significant (Two-way Anova; *p* < 0.05). The increase in TVB−N values for chilled samples may be related to the degradation of protein due to the action of microorganisms and enzymes including deamination and decarboxylation of free amino acids, oxidation of amines and degradation of nucleotides [59,61]. There was no significant difference for TVB−N values from day 1 to day 7 for superchilled samples (*p* > 0.05). A significant increase was observed from day 14 to day 21 of storage for a sample stored at −1.8 °C, while for the samples stored at −2.8 °C and −4 °C there was no significant difference observed for TVB−N values from day 14 to day 21 (*p* > 0.05). Generally, the findings in this study suggest that superchilling might help not only to preserve the quality but also to extend shelf life compared to traditional chilling since the TVB−N values for all superchilled samples remained below the threshold of ≤15 mg/100 g [65] in the whole storage duration.

#### 3.2.6. Changes in TBARS of Beef During Storage

**Figure 8:**
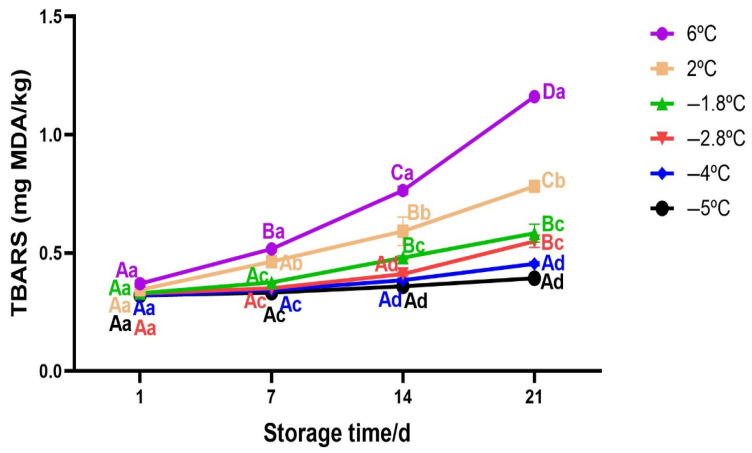
Variation in TBARS of beef meat as a function of storage temperature. Error bars represent the standard error of the mean (SEM) calculated from three replicates (*n* = 3). Note: The data were expressed by the mean ± standard error of the mean. Different superscript lowercase letters in the same storage time indicated the significant difference (*p* < 0.05). Different superscript uppercase letters (A−D) in the same storage temperature indicated the significant difference (*p* < 0.05).

TBARS indicates formation of secondary lipid oxidation products including malonaldehyde (MDA) and other carbonyl compounds which result from hydrolysis of unsaturated fatty acids and are the main contributors of off flavour and off aroma in meat and meat products [18,66]. The results for TBARS are shown in Figure 8. The extent of lipid oxidation was significantly influenced by storage temperature and duration (Two-way Anova; *p* < 0.05). Samples stored at 6 °C exhibited the highest TBARS values, reaching 1.2 mg/kg by day 21, indicating the development of rancid flavour due to lipid oxidation [67]. This may be enhanced by production of lipase enzyme by microbes that hydrolyses fats, releasing free fatty acid which are more prone to oxidative degradation [68]. A similar but less intense trend was observed at 2 °C with a significant increase from 0.4 mg/kg to 0.7 mg/kg. In contrast, samples stored at superchilling temperatures (−1.8 °C and −2.8 °C) showed significantly lower TBARS values throughout storage, with final values remaining below 0.6 mg/kg. Interestingly, TBARS values for samples stored at mild freezing −4 °C and −5 °C remained almost unchanged over the storage period and were among the lowest recorded, suggesting a slowed down of microbial activity. The statistical groupings (different letters) confirm the significant differences among storage conditions and time points. The results obtained at chilling temperatures (2 °C and 6 °C) can be explained by higher extent of production of malondialdehyde and other carbonyl compounds including ketones and aldehydes. Notably, during the analysis, there was unpleasant smell from the meat samples right from day 14 of storage for 6 °C and 2 °C to day 21 of storage suggesting that meat was already not suitable for consumption. This was due to microbial growth as suggested by TVB-N values (see Figure 7) and higher pH (see Figure 4) These observation are in consistent with [69] who performed sensory analysis for chilled pork chops and observed unpleasant smell from trained sensory panellist for TBARS values exceeding 0.6 mg/kg. The results described in the present work highlight the effectiveness of superchilling temperatures in limiting oxidative spoilage, thereby contributing to the preservation of product quality, compared to chilling temperatures.

#### 3.2.7. Changes in Colour

Colour plays a significant role in the consumer acceptability of the meat since it is the main indicator for a fresh meat [70]. Table 2 shows evolution of colour parameters (Lightness L*, redness a* and yellowness b*) during superchilled storage. The initial colour values (L*, a*, b*) of fresh beef samples were 41.62, 27.62 and 19.45 similar to the previous results for fresh beef meat [55,71]. It can be seen from the table that the colour of chilled samples reduced significantly with the increase in storage time (*p* < 0.05). At 6 °C, L* values reduced sharply from 36.06 to 25.78, suggesting beef darkening, while a* values reduced from 20.24 to 7.44, indicating a loss of beef redness. Similarly, b* values declined, reflecting decreased beef yellowness. A similar significant reduction was observed for samples stored at 2 °C but less pronounced in samples stored at superchilling temperatures of −1.8 °C and −2.8 °C. This significant reduction can be related to myoglobin oxidation to metmyoglobin (MetMb) which is dark in colour and lead to visible loss of red colour [72]. However, at lower temperatures −4 °C and −5 °C, L*, a*, and b* values were better preserved. These results suggest that superchilling could significantly slowed the decrease in L*, a*, b* compared to chilled storage [18]. A similar trend was observed for beef meat by [55], where samples stored at −1.5 °C had higher lightness values (L*) compared with samples stored at 4 °C. This confirms that lower temperatures can effectively maintain the colour characteristics of beef over time. On the other hand, it was generally interesting that the redness of the meat a* under chilled condition decreased faster compared to yellowness b*. A similar observation was reported by [73]. They noted that the redness of superchilled beef steaks stored for 16 to 20 weeks was close to the unacceptability threshold of a ≤ 14.5 and this implied poor colour stability over time. The fast decrease in a* was due to oxidation of myoglobin but also higher microbial growth rates at chilling temperature as evidenced by higher TVB-N values (see Figure 7). Moreover, a study by [18] reported that the decrease in a* was related to increase in TBARS formation (also see Figure 8). This suggested that the oxidation of lipids contributes to the accelerated oxidation of myoglobin which aligned also with previous related study [45]. Similarly, [28,74] reported a fast decrease in yellowness b* in rabbit and beef meat, respectively, particularly at higher storage temperatures. This study also found a significant interaction between storage temperature and storage time for the L* value (lightness) (Two−way Anova; *p* < 0.05), suggesting that both factors strongly influenced the change in L*. However, there was no significant interaction for a* and b* values, indicating that their changes were independently influenced by storage temperature and time. These observation align well with the findings of [36] for chicken breast meat.

**Figure 9:**
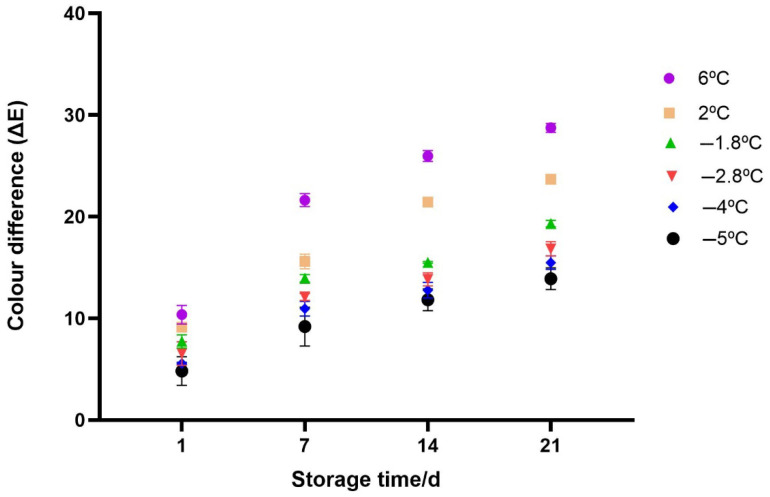
Total colour difference (∆E) for the samples stored under mild-freezing, superchilling and chilling conditions. Error bars represent the standard error of the mean (SEM) calculated from three replicates (*n* = 3).

The total colour difference (ΔE) between the fresh sample and the other samples stored under both chilled and superchilled samples was analyzed (as shown in Figure 9). The values ranged from 4.8 to 28.7 whereas the highest value was observed at day 21 for a chilled sample stored at 6 °C and the lowest value was noticed at day 1 for a sample stored at −5 °C. The general increase in ΔE for all samples, indicated a progressive colour degradation likely due to pigment oxidation as mentioned earlier. In contrast, samples stored at superchilling temperatures (−1.8 °C and −2.8 °C) showed markedly lower ΔE values, particularly at later time points, indicating improved preservation of visual quality. The lowest ΔE values were recorded at −4 °C and −5 °C, suggesting that these lower temperatures effectively minimized colour changes. The change in colour was influenced by both storage temperature and time (Two−way Anova; *p* < 0.05).

### 3.3. Kinetic Modelling of Quality Change

Table 3 shows the kinetic model parameters for selected quality indices which were fitted using Equation (5) for zero order and Equation (6) for first order. The rate constant *k* was obtained from a slope of (*C_t_−C_o_*) Vs *t* and *ln*$\frac{C}{C_{o}}$ Vs *t*, respectively. ${\;R}_{1}^{2}$ is their kinetic rate of coefficient of determination. By fitting the data to Equation (7), the slope of the regression line, the activation energy of the quality degradation reaction $E_{a}$ and the correlation coefficient $R^{2}\;$ describing the temperature dependence of the quality degradation reaction rate constant $k_{d}$ were obtained.

#### 3.3.1. Kinetic Model Parameters

It can be seen from Table 3 that the rate constants for drip loss increased with the decrease in storage temperature as similarly reported by [75] for grounded beef. In addition, the correlation coefficient ${(R}^{2})$ fitted from Arrhenius model was 0.9974 suggesting that a zero−order kinetic reaction could be used for drip loss modelling at different storage temperatures. For colour difference (∆E), TBARS and TVB−N, the trend looks similar as their rate constants increased with the increase in storage temperature. On the basis of Arrhenius equation, their fitting correlation coefficients $R^{2}$ were 0.9906, 0.9458 and 0.9923, respectively. These results suggest that the rate of change in these quality parameters can be modelled using zero kinetic order due to strong correlation between the rate constant and storage temperature. The firmness of beef was described using the first order kinetic reaction and the $R^{2}$ was 0.7439. In addition, the rate constant of firmness degradation decreases with the decrease in storage temperature. These results suggest that a first order kinetic model may be a good fit for predicting degradation of beef firmness. The value for activation energies $E_{a}$ for drip loss, texture, TVB−N, TBARS and colour difference (∆E) were calculated as 110.111, 52.870, 68.553, 119.480 and 47.301 kJ/mol, respectively. Since the activation energy is the energy required to start the reaction and it can be seen that $E_{a}$ for colour difference (∆E) is the lowest among all. This implies that colour degradation occurs more easily under aerobic conditions compared to other reactions [72]. Moreover, the $E_{a}$ for TVB−N is less than that of TBARS this implies that protein degradation is more likely to occur than lipid oxidation since they require less activation energy. In fact, this made low temperature storages to be more effective in slowing down lipid oxidation than protein degradation. These results are in contrary to those reported for rabbit meat but in frozen storage of −18 °C [28].

**Figure 10:**
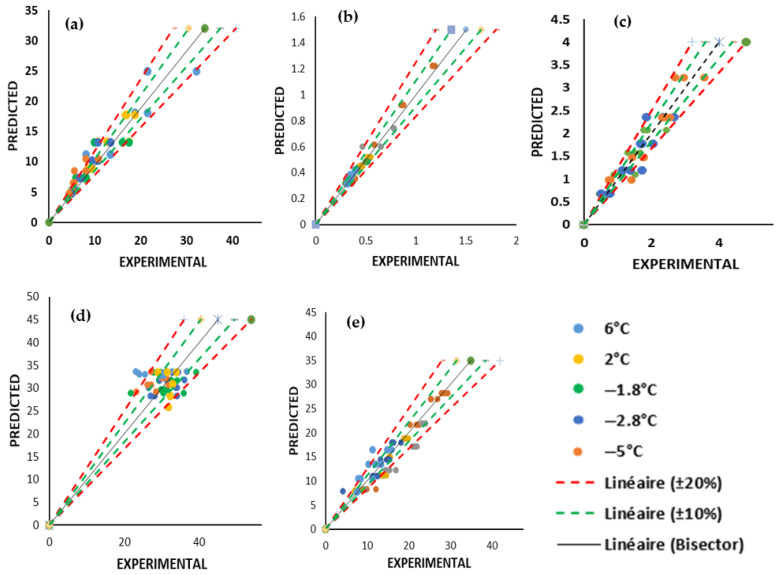
Scatter plots of predicted and experimental quality parameters during 21 days of storage. (**a**) TVB−N; (**b**) TBARS; (**c**) Drip loss; (**d**) Texture; (**e**) Colour difference.

The results of our findings show that the coefficient of determination ${\;R}_{1}^{2}$ for drip loss, colour difference, TBARS and TVB−N were above 0.90. Therefore, this clearly suggests a strong correlation between predicted and experimental values as seen from the scatter plots on Figure 10. The difference between model predictions and experimental data were within ±10% for the majority of data points, with minimum deviations exceeding ±20%. In addition, the observed deviation between the predicted and experimental values was within the range of experimental standard mean error. This suggests that degradation of these quality parameters can be accurately predicted. However, it can be seen that for texture parameter, 20 out of 80 data points were out of ±10% and its ${\;R}_{1}^{2}$ was 0.74 showing a weaker fit. This implies that there are other factors such as microstructural changes that might cause degradation of firmness which cannot be readily captured by the kinetic model.

#### 3.3.2. Model Validation

**Figure 11:**
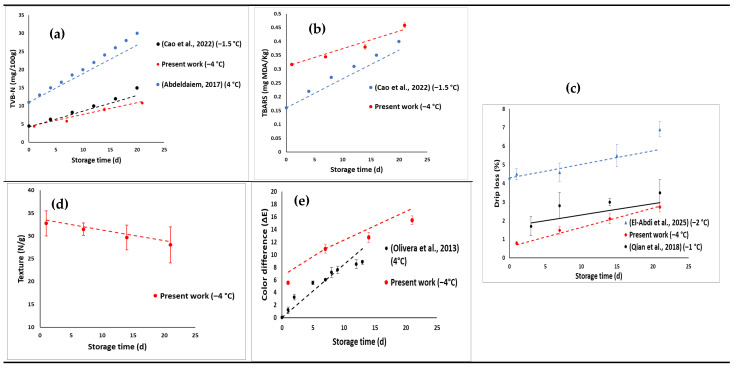
Validation of model prediction for different quality parameters. The dotted line represents the experimental results obtained from this study at −4 °C and from literature (Storage temperature indicated on the figure) while the full line represents the model results: (**a**) TVB−N; (**b**) TBARS; (**c**) drip loss; (**d**) texture; (**e**) colour difference [23,55,72,76,77].

Figure 11 illustrates the validation of the kinetic models developed from this study. The validation was performed by predicting the quality change in beef samples stored at −4 °C as previously described. In addition, data from the literature were also used to compare with the developed kinetic models in the present work in order to assess their accuracy and applicability. Generally, the results show a strong agreement between model predictions and experimental data of the present work for beef stored at −4 °C across all the key quality parameters including TVB-N (Figure 11a), TBARS (Figure 11b), drip loss (Figure 11c), texture (Figure 11d), and colour difference (ΔE) (Figure 11e). On the other hand, comparison between model predictions and literature values also showed good alignment for TVB-N and TBARS of beef stored at −1.5 °C [55] (Figure 11a,b) and at 4 °C [76] (Figure 11a). For drip loss (Figure 11c), the model predictions were inconsistent with literature values reported for beef stored at −1 °C [77] and for chicken stored at −2 °C [23]. This highlights the applicability of the model to predict drip loss in other meat products beyond beef meat. Finally, the model developed accurately predicted colour difference (ΔE), showing good alignment with the values reported by [72] for beef at 4 °C. These results show that the established models were able to provide a realistic prediction of the change in quality over time within a range of 6 °C to −5 °C. For instance, all the quality parameters both from this study and literature sources, the MAPE were within ± 10% suggesting the accuracy of the model (Table 4). Similarly, low values of RMSE were obtained indicating the robustness of the model [78]. However, the prediction of drip loss and colour difference from literature [72,77] showed larger MAPE above 10% for beef (Table 4). The large MAPE might be due to experimental variability and differences in raw material (particular section of the cow) which were not accounted for when modelling.

## 4. Conclusions

This study evaluated quality changes for superchilled beef under two different superchilled storage temperature conditions (−1.8 °C and −2.8 °C) as compared with mild-freezing (−4 °C and −5 °C) and chilling (2 °C and 6 °C) storage conditions. The results obtained provide substantial evidence that partial freezing had minimal effects on beef quality. Moreover, superchilling storage significantly slowed the deterioration of beef quality, particularly in terms of colour, texture, TBARS, TVB-N, drip loss and pH. However, beef samples stored at chilled temperatures particularly at 6 °C and 2 °C showed significantly more pronounced quality deterioration, indicating that higher temperatures accelerate microbial spoilage. For instance, at the end of storage, the TVB-N values of beef stored under 6 °C and 2 °C exceeded the spoilage threshold limit of 15 mg/100 g, while those stored at (−1.8 °C and −2.8 °C) and (−4 °C and −5 °C) remained below this limit. Samples at −1.8 °C and −2.8 °C maintained their firmness throughout the whole storage duration. Mild-freezing temperatures (−4 °C and −5 °C) caused more structural damage and loss of firmness due to ice crystal formation. However, the firmness at 6 °C and 2 °C were the lowest among all. Therefore, among all storage methods, storage at −1.8 °C and −2.8 °C offers the best compromise between quality preservation, energy use and muscle integrity preservation.

Furthermore, this study successfully developed and validated kinetic models to describe the behaviour of key quality indicators during storage. The models demonstrated strong predictive performance, with RMSE and MAPE values within ±10%. These models can support cold chain stakeholders—including food manufacturers, supply chain managers and quality control personnel—in optimizing storage conditions, minimizing spoilage and maintaining product quality. Future work will focus on integrating kinetic models into the ENOUGH tool [79] which is a software for simulating cold chains with respect to quality of products, energy use and CO_2_ emission (environmental) impact of superchilling technology. To strengthen future research, microbiological assessment should be considered to provide a more comprehensive evaluation. In addition, further studies should also focus on linking microstructural changes with measurable quality parameters to better understand the mechanisms underlying meat quality evolution during superchilling storage.

## Figures and Tables

**Table 1:** Average product temperatures under storage conditions (mean ± standard error).

Set Temperature (°C)	Measured Temperature (°C)				Average Measured Temperature (°C)
	Day 1	Day 7	Day 14	Day 21	
−5	−5.1	−5.1	−4.7	−5.1	−5.1 ± 0.2
−4	−4.2	−4.2	−4.1	−4.1	−4.2 ± 0.1
−2.8	−2.75	−2.9	−2.85	−2.8	−2.8 ± 0.1
−1.8	−1.76	−1.7	−1.8	−1.79	−1.8 ± 0.1
+2	2.0	2.0	2.5	2.0	2.1 ± 0.2
+6	5.8	6.2	6.1	6.1	6.1 ± 0.2

**Table 2:** Effects of mild-frozen, superchilled and chilled storage on the lightness (L*), redness (a*) and yellowness (b*) of beef (mean ± standard error).

Storage Time/Days	Storage Temperature (°C)	Colour Parameters		
		L*	a*	b*
1	6.0	36.06 ± 0.34 ^Aa^	20.24 ± 0.91 ^Aa^	14.77 ± 0.40 ^Aa^
	2.0	37.24± 0.15 ^Aa^	20.81 ± 0.35 ^Aa^	15.42 ± 0.36 ^Aa^
	−1.8	38.48± 0.56 ^Ab^	21.65± 0.34 ^Ab^	15.26± 0.55 ^Aa^
	−2.8	39.63 ± 0.76 ^Ab^	22.27± 0.91 ^Ab^	16.25± 0.52 ^Ab^
	−4.0	40.65 ± 0.18 ^Ab^	22.86 ± 0.24 ^Aab^	16.74 ± 0.18 ^Ab^
	−5.0	41.15 ± 1.34 ^Ab^	23.34 ± 1.06 ^Ac^	17.29 ± 0.98 ^Ab^
7	6.0	29.24 ± 0.36 ^Ba^	12.03 ± 0.49 ^Ba^	11.05 ± 0.57 ^Ba^
	2.0	33.50 ± 1.06 ^Bb^	16.66 ± 0.28 ^Bb^	11.90 ± 0.28 ^Ba^
	−1.8	34.38 ± 0.41 ^Bb^	17.62 ± 0.74 ^Bb^	14.49 ± 0.14 ^Bb^
	−2.8	35.60 ± 0.61 ^Ab^	18.58 ± 0.13 ^Bb^	13.73± 0.72 ^Bb^
	−4.0	37.57 ± 0.18 ^Bc^	18.75 ± 0.77 ^Bb^	14.32 ± 0.23 ^Bb^
	−5.0	38.89 ± 0.13 ^Bc^	19.64 ± 1.11 ^Bc^	14.80 ± 0.38 ^Bb^
14	6.0	27.86 ± 0.74 ^Ca^	8.69 ± 0.40 ^Ca^	9.39 ± 0.15 ^Ca^
	2.0	31.40 ± 0.94 ^Bb^	11.71 ± 0.26 ^Cb^	10.32 ± 0.42 ^Bb^
	−1.8	34.01 ± 0.40 ^Bc^	16.54 ± 0.24 ^Cc^	13.32 ± 0.72 ^Bb^
	−2.8	34.89 ± 0.08 ^Bc^	17.10 ± 0.71 ^Bc^	13.49± 0.60 ^Bb^
	−4.0	35.27 ± 0.05 ^Cc^	17.81 ± 0.67 ^Bc^	14.34 ± 0.66 ^Bb^
	−5.0	36.16 ± 0.26 ^Cc^	18.14 ± 0.81 ^Bc^	14.99 ± 0.88 ^Bb^
21	6.0	25.78 ± 0.27 ^Da^	7.44 ± 0.23 ^Da^	6.52 ± 0.06 ^Da^
	2.0	30.01 ± 0.57 ^Bb^	10.03 ± 0.27 ^Db^	8.67 ± 0.02 ^Cb^
	−1.8	31.79 ± 0.59 ^Cb^	13.10 ± 0.22 ^Db^	12.67 ± 0.27 ^Cc^
	−2.8	34.28 ± 0.81 ^Bc^	13.78 ± 0.46 ^Cb^	13.97 ± 0.55 ^Bc^
	−4.0	34.94 ± 0.31 ^Cc^	14.67 ± 0.75 ^Cb^	13.29 ± 0.62 ^Cc^
	−5.0	36.12 ± 0.21 ^Cd^	16.07 ± 1.57 ^Cc^	14.57 ± 0.47 ^Bc^

Notes: Different superscript lowercase letters in the same storage time indicated the significant difference (*p* < 0.05). Different superscript uppercase letters (A–D) in the same storage temperature group indicated the significant difference (*p* < 0.05).

**Table 3:** Kinetic model parameters.

Quality Indicator	Storage Temperature (°C)	Kinetic Parameters				
		k	${\;R}_{1}^{2}$	$E_{a}$ (kJ/mol)	$R^{2}$	$K_{d}$ $({day}^{-1}$)
Drip loss	−1.8	0.068	0.9946	110.111	0.9974	4.4 × $10^{-23}$
	−2.8	0.085	0.9887			
	−5	0.1238	0.989			
Texture	6	0.019	0.9744	52.870	0.7439	1.4 × $10^{8}$
	2	0.015	0.9711			
	−1.8	0.007	0.9627			
	−2.8	0.006	0.9782			
	−5	0.002	0.9703			
TVB−N	6	0.985	0.9988	68.553	0.9923	6.6 × $10^{12}$
	2	0.604	0.9921			
	−1.8	0.441	0.9978			
	−2.8	0.378	0.9977			
	−5	0.280	0.9808			
TBARS	6	0.0409	0.9984	119.480	0.9458	9.9 × $10^{20}$
	2	0.0218	0.9916			
	−1.8	0.0125	0.9978			
	−2.8	0.0098	0.9561			
	−5	0.004	0.9713			
Colour difference	6	0.9864	0.9508	47.301	0.9906	7.0 × $10^{8}$
	2	0.7334	0.9499			
	−1.8	0.5372	0.9295			
	−2.8	0.4818	0.9311			
	−5	0.4424	0.9559			

**Table 4:** Evaluation of the model performance using RMSE and MAPE.

Quality Parameter	Source	RMSE	MAPE (%)
TVB−N	Present work	0.49	6.98
	[55]	0.81	8.17
	[76]	1.52	7.03
TBARS	Present work	0.07	2.79
	[55]	0.13	6.7
Drip loss	Present work	0.47	5.03
	[77]	0.61	16.31
Texture	Present work	0.26	0.77
Colour difference	Present work	1.4	10.67
	[72]	1.13	16.86

## Data Availability

The research data supporting this study can be found by contacting the corresponding author.

## Nomenclature

$E_{a}$	Activation energy (J${\cdot mol}^{-1})$
k	Kinetic rate constant for quality change
n	Number of observations
C	Measured value of quality parameter at time t
$C_{o}$	Initial value of quality parameter
$C_{obs}$	Observed value of quality parameter at each time point
$C_{pre}$	Predicted value for each observation
R	Universal gas constant (8.314 J$\cdot {mol}^{-1}K^{-1}$)
RMSE	Root mean square error
MAPE	Mean absolute percentage error
t	Storage time (day)
T	Temperature (K)
GDP	Gross domestic product
$t_{pf}$	Partial freezing time (seconds)
$T_{f}$	Initial freezing point in °C
$T_{a}$	Air temperature in °C
ρ	Density of beef (kg/m^3^)
$L_{0}$	Latent heat of fusion of water (333.6 kJ/kg)
P	Shape factor (16 for infinite cylinder)
Q	Shape factor (4 for infinite cylinder)
$h_{a}$	Heat transfer coefficient (W/m^2^K)
K	Thermal conductivity (W/mK)
L*	CIE coordinate for lightness of beef
a*	CIE coordinate for redness of beef
b*	CIE coordinate for yellowness of beef
TBARS	Thiobarbituric acid−reactive substances content
TVB−N	Total volatile nitrogen basic compounds
TBA	Thiobarbituric acid
TCA	Trichloroacetic acid
